# Social Interactivity in Live Video Experiences Reduces Loneliness

**DOI:** 10.3389/fdgth.2022.859849

**Published:** 2022-03-25

**Authors:** Benjamin T. Kaveladze, Robert R. Morris, Rosa Victoria Dimitrova-Gammeltoft, Amit Goldenberg, James J. Gross, Judd Antin, Melissa Sandgren, Melissa C. Thomas-Hunt

**Affiliations:** ^1^Department of Psychological Science, University of California, Irvine, Irvine, CA, United States; ^2^Airbnb, San Francisco, CA, United States; ^3^Koko, San Francisco, CA, United States; ^4^Galaxies and Cosmology Department, Institute of Astronomy, Bulgarian Academy of Sciences, Sofia, Bulgaria; ^5^Negotiation, Organizations & Markets Unit, Harvard Business School, Harvard University, Cambridge, MA, United States; ^6^Department of Psychology, Stanford University, Stanford, CA, United States; ^7^Darden School of Business, University of Virginia, Charlottesville, VA, United States

**Keywords:** loneliness, social connection, internet, internet-mediated communication, experiment

## Abstract

**Background:**

Loneliness, especially when chronic, can substantially reduce one's quality of life. However, positive social experiences might help to break cycles of loneliness by promoting more prosocial cognitions and behaviors. Internet-mediated live video communication platforms (eg Zoom and Twitch) may offer an engaging and accessible medium to deliver such social experiences to people at scale. Despite these platforms' widespread use, there is a lack of research into how their socially interactive elements affect users' feelings of loneliness and connection.

**Objective:**

We aimed to experimentally evaluate whether socially interactivity in live video experience improves loneliness-related outcomes.

**Materials and Methods:**

We recruited participants from an online survey recruitment platform and assigned half to participate in a socially interactive live video experience with 6–12 strangers and the other half to a non-interactive control experience that was designed to be identical in every way but not socially interactive. Participants completed several baseline self-report measures of psychosocial wellbeing, participated in the hour-long video experience (an entertaining astronomy lesson), and then completed some baseline measures again. Four weeks later, we followed up with participants to evaluate their change in trait loneliness since baseline. We Pre-registered our hypotheses and analysis plan and provide our data, analysis code, and study materials online.

**Results:**

Two hundred and forty-nine participants completed the initial study and met inclusion criteria, 199 of whom also completed the 4-week follow-up. Consistent with our predictions, we found that directly after the more socially interactive experience, participants' feelings of connectedness increased more (*p* < 0.001), positive affect increased more (*p* = 0.002), feelings of loneliness decreased more (*p* < 0.001), social threat decreased more (*p* = 0.006), and negative affect decreased more (*p* = 0.003) than they did after the less interactive experience. However, change in trait loneliness between baseline and 4 weeks later did not differ between conditions (*p* = 0.953).

**Conclusions:**

Including socially interactive components in live video experiences can improve loneliness-related psychosocial outcomes for a short time. Future work should explore leveraging these benefits toward longer-term prosociality. Future work can also identify if the effects we observed generalize across different populations and kinds of online experiences.

## Introduction

Loneliness, a painful emotional state caused by perceived social isolation, is a ubiquitous part of the human experience (1). In small doses, the gnaw of loneliness can be adaptive, motivating us to exert effort to maintain and seek out high-quality social relationships. Yet, when loneliness becomes chronic it can profoundly reduce wellbeing, making us miserable and putting us at risk for disease (2, 3). Chronic loneliness is strongly associated with social anxiety, depression severity, suicidality, and health-related behaviors, and it predicts mortality to an extent comparable to physical inactivity and lack of access to healthcare (4–6). Loneliness is also extremely common: 20% of Americans surveyed in 2008 reported feeling sufficiently isolated for it to be a major source of unhappiness in their lives (4) and nearly half of 20,000 Americans surveyed in 2018 reported sometimes or always feeling that no one knew them very well (7). The prevalence of loneliness is relatively similar in other Western nations (8) and has remained fairly stable over the past several decades (9, 10).

Experiencing chronic loneliness shifts one's perceptions, motivations, and emotions away from prosociality, impairing functioning and paradoxically making it harder to form high-quality social relationships (5). To address these impairments, evidence-based loneliness interventions generally focus on building social skills, improving connection to high-quality social relationships, and challenging maladaptive social cognitions (11). However, randomized controlled trials of these interventions (11, 12) generally demonstrate weaker effects than most other social and behavioral interventions (13). In addition, these interventions tend to focus on individuals, rather than populations or systems. Thus, new strategies for addressing loneliness at scale may be needed.

The need to help people struggling with loneliness is particularly evident during the COVID-19 pandemic. Although mean levels of loneliness have not changed significantly in either the United States (14, 15) or the United Kingdom (16) during the pandemic, certain groups (such as college students) have experienced disproportionately large increases in loneliness (17, 18). Work from early in the COVID-19 pandemic found that lonely individuals were twice as likely as Non-lonely individuals to be worried about the pandemic negatively impacting their wellbeing (16) and 82% more likely to experience depression-anxiety comorbidities during the pandemic (19).

Fortunately, new behaviors related to internet-mediated communication that emerged during the pandemic may hold potential to help counteract loneliness (20). In particular, live streaming is a form of internet-mediated experience that has grown significantly in popularity since the COVID-19 pandemic began, attracting large global audiences (21). Live streams are internet-mediated experiences in which users watch an audiovisual presentation and, in some cases, can interact with other viewers and the streamer (i.e., the person presenting) in real-time via a text chat. Several studies have demonstrated these streams' relevance to viewers' feelings of connection and community. For example, a qualitative study of mental health discussion on a major live streaming platform, Twitch (twitch.com), noted the potential for interactivity in live streams to provide users a valuable sense of belonging to a community (22). Another study found that viewers who actively participate in text chat during live streams who form “parasocial relationships” with Twitch streamers are more likely to enjoy live streams (23). Finally, an experiment found that when streamers reacted to messages from viewers and addressed viewers individually, viewers enjoyed the live stream more and became more committed to following social norms within the stream (24). Yet, there is a lack of empirical work directly evaluating live streams' impacts on loneliness or other aspects of user wellbeing.

In response to the COVID-19 pandemic, many companies embraced the affordances of video chat and developed new products to help people connect over live video. Airbnb, for example, created a video chat product that allowed would-be travelers to engage in activities and cultural experiences across the world using Zoom (a popular video chat and video conferencing platform). The product is called “Online Experiences” and was developed to simulate elements of local tourism through interactive virtual walking tours (led and designed by local tour guides), cooking shows, and science lessons from around the world (25). It builds from established online formats, such as Webinars (26) or live streams such as Twitch or YouTube Live (27), but was designed to promote deeper levels of social interactivity and connection. To promote this interactivity, these sessions impose a limited group size (28) and emphasize webcam interactions between audience members and the presenters (29). However, it is unclear whether this socially interactive format offers a deeper sense of connectedness or greater benefit to wellbeing than more passive viewing experiences.

In this study, we tested our expectation that participation in a socially interactive live video experience could improve loneliness-related psychosocial outcomes. In particular, we conducted an experiment to test whether a live stream experience with features that aim to foster social interaction among participants would impact feelings of loneliness, connectedness, social threat, and affect (positive and negative) differently than a live stream experience without these social elements. Our main hypotheses were that, compared to participants assigned to the non-interactive experience, participants assigned to the socially interactive experience would report 1) a greater decrease in state loneliness Post-session, 2) a greater increase in social connectedness Post-session, 3) a greater decrease in vigilance to social threat Post-session, 4) a greater increase in positive affect Post-session, 5) A greater decrease in negative affect Post-session, and 6) a greater decrease in trait loneliness 4 weeks after the session.

## Materials and Methods

### Participants

We recruited study participants from Prolific, an online platform for recruiting participants for scientific experiments (30), from July 6, 2021 to August 27, 2021. As inclusion criteria to begin the study, we required participants to have never participated in the pilot version of the experiment, have access to a desktop/laptop computer, have access to Zoom software and be willing to turn on the camera and audio, be at least 18 years of age, and speak English as their first language. We paid each participant $11.51 through Prolific for successfully completing the initial survey, choosing to exceed Prolific's minimum payment of $6.50 per hour for purposes of fair pay.

We recruited participants from Prolific in waves scheduled for specific times based on the availability of the research team member hosting all of the online presentations (RDG). Each wave included one control group and one experimental group, and in each wave both groups' online presentations began at the same time to avoid time-linked confounders. We also recruited each wave to ensure that each group had at least 6 participants and at most 12.

To randomly allocate participants to one condition, we created recruitment posts on Prolific such that half of the posts assigned participants to the experimental condition and the other half assigned participants to the control condition once they enrolled in the study. To reduce the likelihood of expectation effects and willingness to participate causing differences across conditions at baseline, we did not inform participants that two different conditions existed. All details in the study posting (the title, description, eligibility criteria, and remuneration) were identical for both conditions. Because both study posts in a given wave were scheduled in advance for the same date and time, they appeared alongside each other in the list of studies on Prolific. We randomized their position in the Prolific queue by appending a random 6-digit ID to each title. This ensured there was no systematic ordering effect, such that one condition was always presented before the other in the queue. Each Prolific post and consent form explained that participants should only enroll in the study if they were willing to participate in an interactive hour-long Zoom presentation for which they might be asked to turn on their webcam and actively participate.

### Procedure

Our experiment compared change in participants' self-reported loneliness-related psychosocial outcomes across two conditions: the experimental condition was a socially interactive live video experience designed to mimic Airbnb's Online Experiences, and the control condition was intended to mirror the experimental condition in every way except for the live social interaction elements.

Immediately after enrolling in the study and affirmatively responding to an informed consent form, participants completed a baseline survey (described below). Next, participants were given a link to join an online presentation and instructed to return to the survey only after the presentation was finished. Both the control and experimental condition presentations were set to last 60 min.

In the experimental group, the presenter (RDG), an astronomer and science communicator, gave a presentation, titled “Learn About Space with an Astronomer”. On-screen, participants saw the presenter, the presentation slides, and the other study participants. In each session, the presenter greeted each audience member, asked questions periodically of the wider group, and encouraged active participation and interaction between participants. Participants were able to interact by voice and chat and cameras were turned on during the sessions.

In the control group, participants viewed a Pre-recorded presentation from the same presenter that was intended to be as close as possible to the live presentation, except that participants did not have a chance to interact with or see the other study participants (participants were asked to turn on their webcams anyway). Although the control condition was Pre-recorded, participants were led to believe it was a live presentation. To help simulate a live experience, participants were allowed to write messages to the presenter and the presenter answered Pre-scripted questions as if the questions were coming from an audience in real-time.

Immediately after the presentation, individuals in both conditions were instructed to return to the survey to complete Post-session measures, which included all the measures assessed at baseline except for the trait loneliness measure. The Post-session measures also included single items evaluating perceptions of the presenter's friendliness, expertise, and enthusiasm, as well as two attention check questions. Four weeks later, we reached out to participants who completed the original survey via email to ask them to complete a 2-min reassessment of their trait loneliness.

### Materials

All study materials, including a recording of the control condition presentation and the initial and follow-up surveys, are available online (31).

#### Dependent Variables

Before and directly after the online presentation, we first measured positive and negative affect using the 10-item Positive and Negative Affect Schedule (32). Second, we asked participants to indicate how lonely they felt in the present moment, from 1 (Not at all) to 5 (Extremely). Third, we asked them to report how connected they felt to other people in the present moment, from 1 (Not at all) to 5 (Extremely). Fourth, we measured hypervigilance to social threat by asking participants to indicate how much criticism or rejection they expected from their next social interaction from 1 (Not at all) to 5 (Extremely). Finally, we evaluated trait loneliness using Version 3 of the 20-item UCLA loneliness scale (33).

#### Independent Variables

We included two dummy-coded binary independent variables: condition (0 = experimental, 1 = control) and time (0 = Pre-presentation, 1 = Post-presentation).

#### Demographics

Participants were asked to self-report the following demographic variables: age (continuous), gender identity (categorical, with levels male, female, Non-binary, other, and prefer not to answer), country of residence (open response), and first language (open response).

#### Manipulation Checks

To ensure that participants' perceptions of the presentation content were similar across conditions, we asked participants in both conditions to rate how friendly the presenter was, how much expertise the presenter had, and how much enthusiasm the presenter had (all three rated from 1 [None at all]−5 [Extremely]). As a manipulation check to test if participants in the control condition believed that the presentation they saw was live, we asked participants at the end of the initial survey to indicate if they had just viewed a “live webinar with other participants” or a “Pre-recorded video” (only a subset of participants saw this item as we decided to include it after beginning data collection).

#### Attention Checks

We included two attention checks, which were multiple-choice questions that were difficult enough that they could not be answered easily through a web browser search, but easy enough to recall for anyone who paid attention during the presentation. For example, “what is the real color of the sun?”. According to the presentation, the correct answer was “green” because that is the predominant wavelength that is emitted by the sun.

### Analysis Plan

Our Pre-registered analysis plan is available online (34), in addition to the analysis code (35) and de-identified dataset (36) to replicate analyses. In the interest of increasing the experiment's power, we used a mixed design, comparing the change in outcomes within participants across time and between participants across conditions. We used a multi-level model for each outcome with condition, time, and the 2-way interaction between condition and time as predictors, participant identifier as a random intercept, and participants nested within each session to account for session-level differences (i.e., a preponderance of extroverts in one group and the time of day of the session). Using the “lme4” package in R, these analyses took the following form:

lmer(outcome ~ time^*^condition + (1|Session_ID/ Participant_ID)) (37).

To test for faulty randomization, differential dropout, and whether participants believed they were in a live presentation, we used chi-squared tests with Yates's continuity correction. To test if perceptions of presenter friendliness, enthusiasm, and expertise were similar across conditions and across dropout, we used *t*-tests assuming unequal variance and Welch's approximation of degrees of freedom. For all analyses, we used the standard *p* < 0.05 criterion for determining statistical significance. Between-subjects analyses were conducted using the *t*-test() and chisq.test() functions within the “stats” package in R (38). All multi-level model analyses were conducted using the lmer() function within the “lme4” package. Finally, we used the “sjPlot” package to create multi-level model output tables and to calculate *p*-values for those models using the Kenward-Rogers approximation (39).

#### Exclusion Criteria

We Pre-registered several exclusion criteria: participants were to be dropped from our main analyses if they failed to answer either of the two attention check questions, if they completed the Post-session questionnaire before completing the presentation as instructed, or if they were in a live session that had more than 12 or fewer than 6 participants. For participants who completed the survey multiple times, we also decided to exclude all survey attempts after their first attempt from analysis, although we did not think to Pre-register this exclusion rule.

## Results

### Participants

Four hundred ninety-three initial surveys (Pre-session, presentation, and Post-session) were completed, although 32 users began the survey twice and one began it 3 times. We first excluded data from 198 of these 493 surveys because their Post-session survey measures were completed before the presentation was completed. Next, we excluded data from 42 surveys because of failure to provide the correct response to either of the two attention check questions. Finally, we dropped 4 surveys from respondents who had already taken the survey. This produced a final dataset of 249 participants who finished the entire initial survey and passed attention checks. Of these 249 participants, 199 also completed the 4-week follow-up survey. All of the presentation sessions fell within the required bounds of 6–12 participants, so all 30 sessions were included in the analyses.

Descriptive statistics among the 249 participants who finished the entire initial survey are shown in Table 1. 42.6% of participants were from the United Kingdom, 35.3% were from the United States, 10.8% were from South Africa, 5.6% were from Canada, and 4.2% were from other countries. The mean (43.53) and standard deviation (11.94) of trait loneliness in this sample were very similar to those of other recent samples of adults around the globe (40). Nearly all participants responded at or near the minimum value of state loneliness and social threat, leaving little room to detect reductions in those variables. Finally, ratings of presenter friendliness, enthusiasm, and expertise were all high.

**Table 1:** Descriptive statistics, all measured at baseline except for presenter friendliness, enthusiasm, and expertise, which were evaluated after the presentation.

**Descriptive statistics of variables**	
**Characteristic**	***N*** **= 249**^***a***^
Age	33.57 (12.06)
Gender	
Male	100 (40.0%)
Female	139 (56.0%)
Non-binary	9 (3.6%)
Chose not to respond	1 (0.4%)
State loneliness (1–5)	1.73 (0.90)
Connectedness (1–5)	2.83 (0.95)
Social threat (1–5)	1.49 (0.81)
Trait loneliness (20–80)	43.53 (11.94)
Positive affect (5–25)	16.39 (4.03)
Negative affect (5–25)	6.76 (2.43)
Presenter friendliness (1–5)	4.76 (0.51)
Presenter enthusiasm (1–5)	4.81 (0.49)
Presenter expertise (1–5)	4.66 (0.63)

a *Mean (SD); n (%)*.

### Preliminary Analyses

#### Manipulation Checks

Mean perceived presenter friendliness was higher in the experimental condition (*M* = 4.89, *SD* = 0.34) than it was in the control condition (*M* = 4.63, *SD* = 0.62), *t*(180) = 4.16, *p* < 0.001. Mean perceived presenter enthusiasm did not significantly differ across conditions (*p* = 0.106). Mean perceived presenter expertise was higher in the experimental condition (*M* = 4.76, *SD* = 0.53) than in the control condition (*M* = 4.55, *SD* = 0.72), *t*(217) = 2.61, *p* = 0.010. Of the 83 participants who were asked, there was no significant difference in the proportion of people who thought they were in a live presentation across the control (83.3%, 35/42, believed it was live) and experimental (97.6%, 40/41, believed it was live) conditions (*p* = 0.068).

#### Randomization and Differential Dropout

Of the 493 surveys that were started, 230 were randomized to the control condition and 263 to the experimental condition, which was not a statistically significant difference (*p* = 0.137). The proportion of participants that completed the initial survey but not the follow-up survey did not significantly differ across conditions (*p* = 0.281).

Among the 249 final participants, mean values of all variables were roughly similar at baseline across conditions (*p* > 0.05 and <10% difference in means), with the exception of gender. The relative frequencies of gender differed across conditions among the 448 participants who started the survey and indicated their gender; 147 women, 94 men, and 3 Non-binary people were randomized to the experimental condition, while 98 women, 98 men, and 8 Non-binary people were randomized to the control condition, X2(*df* = 2, *n* = 448) = 8.65, *p* = 0.013. There was no differential dropout across genders during the initial survey, *p* = 0.245. We do not know why the gender frequencies differed under this randomized setting, but Prolific noted that there were issues with gender allocation throughout our study (for which they reimbursed us). It is possible that some initial attempts to balance gender by Prolific persisted across multiple waves of the study. The different frequencies could have also been a random event, given that our chi-squared test showed there was a 1.3% chance of a difference in frequencies at least as extreme as the one we observed if there were no systematic differences.

Compared to the 210 participants who started the initial survey but who did not complete it or did not pass attention checks, the 249 who completed the initial survey and passed attention checks had lower state loneliness [*t*(360) = 2.18, *p* = 0.030], did not differ in trait loneliness (*p* = 0.683), had lower negative affect [*t*(349) = 2.75, *p* = 0.006], did not differ in positive affect (*p* = 0.097), did not differ in age (*p* = 0.409), and did not differ in gender distribution (*p* = 0.245), all at baseline. The 249 participants who completed the initial survey and passed attention checks also rated the presenter as more friendly [*t*(134) = 6.97, *p* < 0.001], more enthusiastic [*t*(132) = 5.36, *p* < 0.001], and more expert [*t*(146) = 5.10, *p* < 0.001]. We examine the impacts of this differential dropout on the main analyses in Appendix 1. Among those who completed the initial survey and passed attention checks, there were no significant differences in any variables of interest between those who completed the follow-up and those who did not complete it.

### Main Analyses

**Figure 1:**
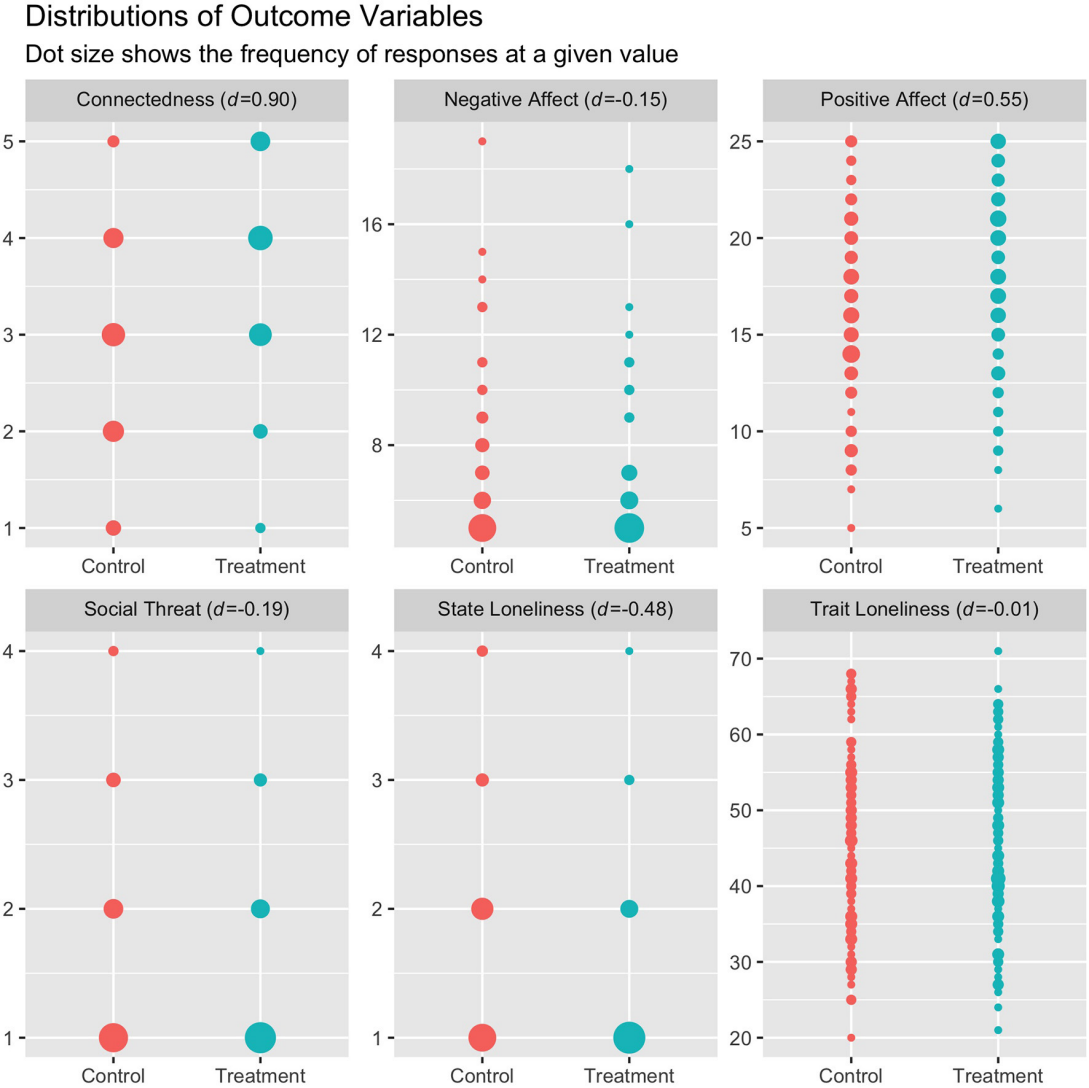
Value frequencies of outcome variables across conditions. We measured outcomes directly after the live video experience for all outcomes except trait loneliness, which we measured 4 weeks later. The sample size was 249 for all outcomes except trait loneliness, which was 199. *d* refers to Cohen's D, measuring the difference in outcome means across conditions at Post-session.

All 5 of our main hypotheses were supported, such that state loneliness, negative affect, and social threat decreased after the presentation to a greater extent in the experimental condition than they did in the control condition, while positive affect and connectedness increased after the presentation to a greater extent in the experimental condition than they did in the control condition (Table 2, Figure 1). These condition-level differences in the association between time point and outcome can be seen in the “time (Post-session): condition (control)” row of Table 2. The change in outcomes between Pre-session and Post-session among individuals in the experimental condition was also significant in all outcomes, as can be seen in the “time (Post-session)” row of Table 2.

**Table 2:** Results of the 5 short-term change hypotheses. State loneliness, negative affect, positive affect, connectedness, and social threat all changed in the predicted directions to greater extents in the experimental condition than in the control condition.

	**State loneliness**			**Negative affect**			**Positive affect**			**Connectedness**			**Social threat**		
***Predictors***	***Estimates***	***CI***	***p***	***Estimates***	***CI***	***p***	***Estimates***	***CI***	***p***	***Estimates***	***CI***	***p***	***Estimates***	***CI***	***p***
Intercept	1.79	1.64 – 1.93	**<0.001**	6.97	6.51 – 7.43	**<0.001**	16.83	15.97 – 17.69	**<0.001**	2.91	2.74 – 3.07	**<0.001**	1.54	1.40 – 1.68	**<0.001**
Time (Post-session)	−0.58	−0.72 – −0.44	**<0.001**	−1.08	−1.42 – −0.73	**<0.001**	1.61	1.02 – 2.21	**<0.001**	0.79	0.62 – 0.96	**<0.001**	−0.25	−0.36 – −0.13	**<0.001**
Condition (control)	−0.10	−0.31 – 0.11	0.344	−0.41	−1.07 – 0.25	0.222	−0.94	−2.17 – 0.30	0.136	−0.17	−0.40 – 0.07	0.171	−0.11	−0.31 – 0.09	0.281
Time (Post-session): condition (control)	0.39	0.18 – 0.60	**<0.001**	0.76	0.27 – 1.25	**0.003**	−1.37	−2.23 – −0.52	**0.002**	−0.69	−0.93 – −0.45	**<0.001**	0.23	0.07 – 0.40	**0.006**
**Random effects**					
σ^2^	0.34			1.97			5.92			0.46			0.22		
τ_00_	0.23 _PID:Presentation_ID_			3.11 _PID:Presentation_ID_			10.29 _PID:Presentation_ID_			0.44 _PID:Presentation_ID_			0.31 _PID:Presentation_ID_		
	0.02 _Presentation_ID_			0.22 _Presentation_ID_			0.97 _Presentation_ID_			0.00 _Presentation_ID_			0.01 _Presentation_ID_		
ICC	0.42			0.63			0.66			NA			0.59		
N	249 _PID_			249 _PID_			249 _PID_			249 _PID_			249 _PID_		
	30 _Presentation_ID_			30 _Presentation_ID_			30 _Presentation_ID_			30 _Presentation_ID_			30 _Presentation_ID_		
Observations	498			498			498			498			498		
Marginal R^2^/Conditional R^2^	0.078/0.466			0.030/0.639			0.055/0.674			0.242/NA			0.015/0.599		

“*PID” refers to participant ID, while “Presentation_ID” refers to the live presentation session in which one participated. Bolded values indicate statistical significance of P < 0.05*.

Our sixth hypothesis, that trait loneliness would decrease 4 weeks after the initial survey to a greater extent in the experimental condition than in the control condition, was not supported, as is shown in Table 3.

**Table 3:** Trait loneliness did not differ between before the presentation and 4 weeks after the presentation to a different extent across conditions.

	**Trait Loneliness 4 Week Follow-up**		
***Predictors***	***Estimates***	***CI***	***p***
Intercept	43.41	41.24–45.58	**<0.001**
Time (Post-session)	1.73	0.47–2.99	**0.007**
Condition (control)	0.34	−2.86–3.53	0.835
Time (Post-session): condition (control)	−0.17	−2.03–1.68	0.853
**Random Effects**			
σ^2^	22.00		
τ_00__PROLIFIC_PID:Presentation_ID_	108.73		
τ_00__Presentation_ID_	0.00		
N _PROLIFIC_PID_	199		
N _Presentation_ID_	30		
Observations	398		
Marginal R^2^/Conditional R^2^	0.031/NA		

*Bolded values indicate statistical significance of P < 0.05*.

### Sensitivity Analyses

Due to the significant differences in gender frequency, perceived presenter friendliness, and perceived presenter expertise across conditions, we conducted sensitivity analyses to examine whether these variables moderated the results of the main analyses. Neither gender, presenter friendliness, or presenter expertise moderated the experimental effect across time for any of the 6 outcomes explored, with the one exception that being in the experimental condition was associated with a smaller reduction in social threat for women than for those identifying as other genders. We also conducted an intent-to-treat analysis, re-running the main analyses with all 459 unique participants who completed at least the first page (consent) of the survey, provided they had data on the relevant variables. The intent-to-treat analysis replicated the findings of the main analysis, except for negative affect and social threat. See Appendix 1 for full details on sensitivity analyses.

## Discussion

Social connection is at the core of human psychology (41). Yet, finding and maintaining high-quality social relationships is often challenging. While everyone experiences loneliness sometimes, chronic loneliness can be destructive to wellbeing (42). Cacioppo & Cacioppo (43) describe loneliness as “a public health problem that can be largely solved in our lifetime”, but effective and scalable loneliness solutions remain elusive (11). If properly leveraged, internet-mediated social technologies may offer a useful medium to improve loneliness-related psychosocial outcomes (44).

Making the most out of social technologies is especially important in the wake of the COVID-19 pandemic, when technology-mediated interactions are central in many people's lives for work, education, entertainment, and social relationships. Thoughtfully designed social technologies may be especially useful for lonely people, who tend to feel more comfortable communicating online than in person (45, 46). Because various kinds of online social experiences have distinct affordances and downsides (47), it is important to identify the kinds of internet-mediated experiences that hold the greatest promise to improve psychosocial outcomes of interest.

In this Pre-registered experiment, we found that participation in a socially interactive live video experience led to greater improvements in several self-rated loneliness-related psychosocial outcomes, immediately after the experience, than participation in a non-interactive control experience did. However, we did not find that this short-term boost led to a greater decrease in trait loneliness 4 weeks later in the experimental condition than in the control condition.

### Decreasing Loneliness

As noted in the introduction, loneliness is a complex phenomenon that is associated with deep cognitive, affective, and behavioral impairments (5). We do not wish to suggest that a single 60-min presentation with interactive social elements is a sufficient loneliness intervention, as evidenced by the lack of a change in trait loneliness 4 weeks after the presentation. However, our evidence does show significant short-term improvements in several loneliness-related outcomes resulting from a socially interactive live video experience. Future work can explore how the short-term boost in affect and prosocial cognitions we demonstrated might translate into longer-term cognitive or behavioral changes. For example, these short interactive experiences could provide a spark of motivation to help people overcome attitudinal barriers to seeking deeper and more frequent social interactions. Indeed, we observed that individuals in the experimental condition showed greater reductions in vigilance to social threat. An openness to engage socially might not lead to enduring effects on its own, but it could make an individual more amenable to social interaction, should the opportunity present itself, which could lead to more self-sustaining cycles of prosociality.

Relatedly, future work should examine whether repeated participation in interactive live video experiences might create stronger and more enduring effects than a single session. It may be that participating in socially interactive live video experiences provides a consistently beneficial boost in psychosocial outcomes, as in receiving a regular “dose” of interpersonal connection. However, it could also be that the initial charm of such live video interactions quickly wears off, so that later exposures are less beneficial. Furthermore, participants in our study had no Pre-existing relationships or expectation of future interaction with the other people in their presentation groups. It may be that online socially interactive experiences impact feelings of connection and loneliness differently when the participants expect to interact again in the future, as in geographically distributed members of the same organization or team (48).

This study offers a novel methodology and conceptual framework that should be built upon to investigate other live video experiences' impacts on social outcomes. Socially interactive live video experiences are woven into a range of popular consumer products–for example, Peloton, in which users receive personalized encouragement from charismatic instructors while competing with other participants in live-streamed exercise classes (49)–and empirical research using designs like ours can examine if such products meaningfully impact users' perceptions of connectedness and community. Our results may also suggest that internet-supported social experiences can supplement more formal loneliness interventions; such experiences could provide low-cost, convenient, and relatively Non-threatening opportunities for individuals to challenge their maladaptive social cognitions and improve their social skills (11, 45). Investigating these questions across different populations and kinds of experiences will help to design more effective socially interactive live video experiences.

### Limitations

First, although we separately evaluate state and trait loneliness, we do not distinguish between transitory and chronic loneliness. This distinction is important because while chronic loneliness is a significant risk factor, transitory loneliness may be adaptive. Second, our single-item state loneliness and social threat measures were not validated and were highly skewed in our sample such that most of our sample did not report struggling with loneliness. This reduced the power of our analyses and impaired our ability to generalize our findings to more lonely populations. Future work should recruit samples who struggle with loneliness to overcome this issue. Third, our use of an online recruitment platform creates some sampling bias; for example, users of these platforms may misreport their age or other characteristics. Sampling bias may have been particularly severe for us because we collected data during a period when the survey platform, Prolific, received a huge influx of new users who were young and mostly female due to a popular post about the platform on the social media site Tiktok (50). Issues with the platform may have also led to a systematic imbalance between our control and experimental conditions. Fourth, our conclusions regarding the live video experience's impacts on loneliness-related outcomes are limited only to the period that we measured them, and there may have been other unintended downsides of participation that we did not think to measure. Finally, we operationalized social interactivity in this study as live video interactions between session participants and with the presenter, but there are many forms of social interactivity. Therefore, our findings need to be replicated across different forms of interaction and platforms.

## Conclusion

This work presented experimental evidence for short-term improvements in state loneliness, affect, connectedness, and perceived social threat as a result of participation in a socially interactive live video experience. This evidence could help to inform the development of technology-supported psychosocial interventions or products that aim to leverage social interaction to improve wellbeing for a general usership.

## Data Availability Statement

The generated datasets for this study can be found at https://osf.io/vthdx/?view_only=None.

## Ethics Statement

The study was reviewed and approved by the Panel on non-medical human subjects, Stanford University. The participants provided electronic consent to participate in the study.

## Author Contributions

RM led pre-registration and data collection. BK performed the statistical analysis and wrote the first draft of the manuscript. All authors contributed to conception and design of the study. All authors contributed to manuscript revision, read, and approved the submitted version.

## Funding

Airbnb funded data collection for this research and paid for open access publication fees.

## Conflict of Interest

During the experimental design, data collection, data analysis, and findings interpretation phases of the study. RM, MS, JA, and MT-H were stockholders and/or paid employees of Airbnb. RD-G is an online experience host on Airbnb and was paid for each study session she conducted. Airbnb covered the cost of the study sessions and the participant incentives. The remaining authors declare that the research was conducted in the absence of any commercial or financial relationships that could be construed as a potential conflict of interest.

## Publisher's Note

All claims expressed in this article are solely those of the authors and do not necessarily represent those of their affiliated organizations, or those of the publisher, the editors and the reviewers. Any product that may be evaluated in this article, or claim that may be made by its manufacturer, is not guaranteed or endorsed by the publisher.

## Appendix 1. Sensitivity Analyses

Data for these analyses are available at (36), and analysis code is available on lines 234–310 of the script at (35).

Gender as a moderator of condition and time

We ran a multi-level model with each of the 6 outcomes. For each model, an outcome was predicted by a 3-way interaction between condition, time (Pre-session/Post-session), and gender as a predictor, with participant identifier as a random intercept and participants nested within their presentation session. Being male, female, Non-binary, or choosing not to report gender did not moderate the interaction between condition and time for any outcome, except for social threat, where being in the experimental condition was associated with a smaller reduction in social threat for women than for those identifying as other genders (*b* = –0.48, *t* = –2.74, *p* = 0.006). Presenter friendliness and expertise as a moderator of condition and time Neither presenter friendliness nor presenter expertise moderated the interaction between condition and time for any outcome.

Intent-to-treat analyses

Re-running the main analyses with all 459 unique participants who completed at least the first page (consent) of the survey, condition moderated the difference between Pre-session and Post-session in state loneliness, *b* = 0.32, *t* = 3.39, *p* = 0.001), not in negative affect (*b* = 0.33, *t* = 1.10, *p* = 0.270), in positive affect (*b* = –1.03, *t* = –2.86, *p* = 0.004), in connectedness (*b* = –0.53, *t* = –4.97, *p* < 0.001), and not in social threat (*b* = 0.13, *t* = 1.49, *p* = 0.135). Like in the main analysis, condition did not moderate the difference in trait loneliness between Pre-session and 4-week follow-up (*b* = 0.05, *t* = 0.06, *p* = 0.953).
